# Uncollected polythene fragments size-dependently alter soil multifunctionality via mediating bacterial drivers in dryland

**DOI:** 10.1093/nsr/nwae281

**Published:** 2024-08-16

**Authors:** Ze-Ying Zhao, Peng-Yang Wang, Xiao-Bin Xiong, Yinglong Chen, Hong-Yan Tao, Wen-Ying Wang, Yajie Song, Muhammad Ashraf, Li Zhu, Yun-Li Xiao, Shi-Sheng Li, Fang-Kun Yang, Meng-Ying Li, Jing Cao, Xiang-Wen Fang, Levis Kavagi, You-Cai Xiong

**Affiliations:** State Key Laboratory of Herbage Improvement and Grassland Agro-ecosystems, College of Ecology, Lanzhou University, China; State Key Laboratory of Herbage Improvement and Grassland Agro-ecosystems, College of Ecology, Lanzhou University, China; State Key Laboratory of Herbage Improvement and Grassland Agro-ecosystems, College of Ecology, Lanzhou University, China; The UWA Institute of Agriculture, and School of Agriculture and Environment, The University of Western Australia, Australia; State Key Laboratory of Herbage Improvement and Grassland Agro-ecosystems, College of Ecology, Lanzhou University, China; Laboratory of Biodiversity Formation Mechanism and Comprehensive Utilization of the Qinghai-Tibet Plateau in Qinghai Province, Qinghai Normal University, China; Global Institute of Eco-environment for Sustainable Development (GIESD), Yale School of the Environment, Yale University, USA; Institute of Molecular Biology and Biotechnology, The University of Lahore, Pakistan; College of Biology and Agricultural Resources, Huanggang Normal University, China; College of Biology and Agricultural Resources, Huanggang Normal University, China; College of Biology and Agricultural Resources, Huanggang Normal University, China; State Key Laboratory of Herbage Improvement and Grassland Agro-ecosystems, College of Ecology, Lanzhou University, China; Laboratory of Biodiversity Formation Mechanism and Comprehensive Utilization of the Qinghai-Tibet Plateau in Qinghai Province, Qinghai Normal University, China; State Key Laboratory of Herbage Improvement and Grassland Agro-ecosystems, College of Ecology, Lanzhou University, China; State Key Laboratory of Herbage Improvement and Grassland Agro-ecosystems, College of Ecology, Lanzhou University, China; State Key Laboratory of Herbage Improvement and Grassland Agro-ecosystems, College of Ecology, Lanzhou University, China; Division of Ecosystems and Biodiversity, United Nations Environment Programme, Kenya; State Key Laboratory of Herbage Improvement and Grassland Agro-ecosystems, College of Ecology, Lanzhou University, China

**Keywords:** uncollectible plastics fragments, soil multifunctionality, bacterial community characteristics, bacterial network, accumulation years

Plastic pollution has emerged as a crucial driver of environmental change [1]. Despite human efforts towards recycling and clean-up, current practice makes it difficult to collect fragments that are <100 cm² in size and this poses a significant challenge for environmental sustainability [2]. Uncollectible fragments might modify soil ecosystem functions via mediating soil microbial communities, particularly soil multifunctionality (SMF) [3–5]. Currently, plastic fragments steadily accumulate in the environment and are believed to have negative impacts on soil function [2,6,7]. However, it remains unclear how the uncollected plastic pollution will affect SMF with increasing accumulation (over the next 30 years) and varying sizes.

To address this widespread concern, a continuous 3-year field investigation was conducted in irrigated dryland. The results showed that various soil functions exhibited differentiate responses to plastic fragments (Fig. 1a–d). Overall, relative to the control group without plastic fragment addition (CK), the nitrogen storage function of the soil was significantly reduced in the area treated with fragment addition. Under low concentrations, nitrogen stocks initially decreased and then increased with increasing fragment sizes (*P* < 0.05). At high concentrations, however, there was no significant trend (Fig. 1a and Fig. S2). On the other hand, the enzymatic activity and DNA concentration functions were markedly enhanced, yet this was highly dependent on the fragment sizes and concentrations (Fig. 1c and e, and Figs S3 and S4). Moreover, medium-sized fragments significantly reduced crop production function across the concentrations. In contrast, under high concentrations, the addition of small fragments (Small-H) evidently promoted crop production function whereas that of large fragments had no pronounced effect (Fig. 1d and Fig. S5). The interactive effects of fragment sizes and concentrations were generally evident on nitrogen stocks and crop production. Finally, the overall carbon storage function was basically unaffected by plastic fragment addition (Fig. 1b and Fig. S6).

**Figure 1.**
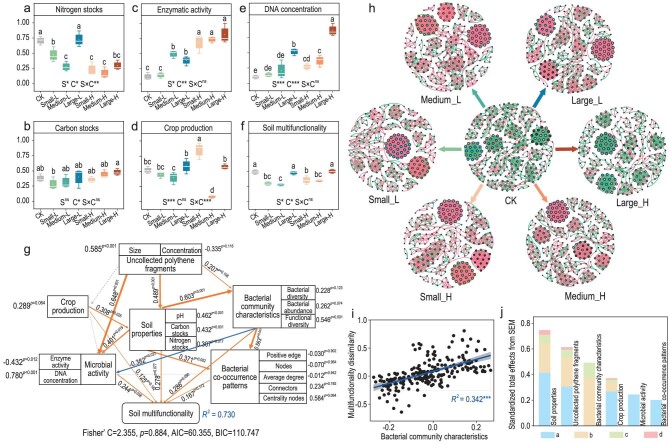
Polythene fragment pollution conditionally alters soil multifunctionality via the mediation of bacterial drivers. (a–f) Effects of plastic fragment pollution on sole soil functions (Z-score) and multifunctionality. (g) Piecewise structural equation model (PiecewiseSEM) accounting for the hypothesized direct and indirect relationships between soil functional groups, bacterial community characteristics (including co-occurrence patterns) and soil multifunctionality. *R*^2^ denotes the proportion of variance explained. (h) Co-occurrence patterns in the soil bacterial network as affected by plastic fragments pollution. The sizes of the nodes (ASVs) are proportional to the number of connections. Only nodes that were significantly correlated with each other (Spearman's > 0.7, *P* < 0.05) were connected (edges). (i) Correlation between dissimilarity in bacterial community characteristics and dissimilarity in soil multifunctionality. (j) The standardized effects (*λ*) of the components in the model on the SMF derived from PiecewiseSEM. a + b + c + d, total effect. b + c, mediating effects of bacterial community characteristics. c + d, mediating effects of bacterial co-occurrence patterns. For each function, data followed by different lower-case letters indicate the significant differences at *P* < 0.05. ***P < 0.001; **P < 0.01; *P < 0.05.

In total, the SMF index was significantly lowered by 33.2% and 35.3% in response to small and medium-sized fragments, respectively, relative to CK (*P* < 0.05). However, there were no significant changes in SMF for large fragments (Fig. 1f). Specifically, compared with large fragments, small and medium-sized fragments exhibited disruptive effects on soil aggregate structure and hydrothermal status (Fig. S7). In particular, medium-sized fragments displayed an absolutely negative impact on plant growth (Fig. S5). The above phenomenon generally aggravated the micro-environmental instability of the soil [8]. Interestingly, a high concentration of fragments generally resulted in higher SMF regardless of size. However, the interactions between sizes and concentrations were not significant (Fig. 1f). A general trend was that the contrasting sizes and concentrations of plastic fragments significantly altered multiple soil ecosystem functions and bacterial community characteristics (Figs S2–S6 and S8–S11). Microbial diversity and soil functionality were positively correlated in both natural and agricultural ecosystems [9,10]. In the plastic-contaminated agricultural soils, the linear regression model showed significant positive correlations between SMF dissimilarity and bacterial community characteristic dissimilarity (*P* < 0.001) (Fig. 1i and Figs. S11 and S12).

On the other hand, increasing plastic residues significantly altered soil bacterial communities, as evidenced by notable changes in co-occurrence patterns (Fig. 1h, Fig. S13 and Table S1). Relative to CK, the average clustering coefficient tended to increase in the groups with plastic fragment addition. However, the number of nodes showed a decreasing trend (Fig. S13). In addition, there was an increasing tendency in either the positive edges or the average degree in the small-fragment and medium-fragment groups, while no significant differences were observed in the large-fragment group relative to CK (Fig. S13). Notably, the small and medium-sized fragments altered the node connectivity by reducing the number of nodes with increased positive edges, resulting in a denser network. This densification may have simplified the co-occurrence patterns, potentially reducing the network complexity and stability [11]. Certainly, the competition among microorganisms enhanced network robustness [12].

To a large extent, the alterations in the network structure represent the ecological adaptive responses of soil microorganisms to plastic fragments [13]. In the present study, the impacts of fragment concentrations on network structures were statistically insignificant but became significant when interacting with different sizes (Table S1). Surprisingly, there was only a slight change in the stability of the network in the large-fragment groups (Fig. S13). Yet, small and medium-sized fragments brought about more pronounced effects on bacterial co-occurrence relationships than did large ones, due to their larger contact area with the soil matrix. Given that SMF could be affected by soil bacterial community characteristics, the associations of SMF with bacterial co-occurrence patterns were also analysed (Figs. S13–S15). There were strong correlations between the topological properties of the network and SMF (Fig. S15). Here, the network centrality node, network connector, positive edge, node number and average degree were identified as the major factors affecting SMF (Fig. S15). It can be argued that the microbial network structure played a crucial role in shaping SMF (Fig. 1j and Fig. S16).

On the other hand, random forest modeling demonstrated that bacterial community characteristics (including co-occurrence patterns) exhibited a greater contribution to SMF relative to nitrogen or carbon storage when considering multiple soil drivers (Fig. S16). Use of the Piecewise structural equation model explained 73% of the variance in SMF. Specifically, the majority of key functional groups displayed positive effects on SMF, including microbial activity (λ = 0.244), soil properties (λ = 0.278), bacterial community characteristics (λ=0.286), bacterial co-occurrence patterns (λ = 0.167) and crop production (λ = 0.125), respectively (Fig. 1g). In total, the characteristics of the bacterial community were as influential as or more influential than the other functional groups, which was consistent with the outcome from the random forest analyses (Fig. 1g and j, and Fig. S16). Mechanistically, the impacts of uncollected plastic fragments on SMF were largely mediated by the soil bacterial community (Fig. 1g, i and j), as the λ values of the bacterial community characteristics and the co-occurrence patterns affecting SMF were up to 0.491 and 0.200, respectively. The mediation analyses demonstrated that bacterial community characteristics explained 47.3% of the relationships between the plastic fragments and the SMF, while co-occurrence patterns only accounted for 12.0% (Fig. 1j). Therefore, the enhancement of bacterial community functions was helpful in mitigating the negative impacts of plastic fragments on soil ecosystems. This trend could result in relatively strong SMF accordingly. Moreover, the sizes (λ = 0.585) and concentrations (λ = –0.335) of uncollected plastic fragments can indirectly affect SMF, as resulted from their positive and negative effects on microbial activities (λ = 0.648), soil properties (λ = 0.489) and bacterial community characteristics (λ = 0.207), respectively (Fig. 1g). Overall, fragment sizes generally generated positive effects on SMF, while concentrations had negative effects on SMF. The effects of the sizes on SMF were more evident than those of the concentrations (Fig. 1g and Fig. S16).

Taken together, there existed significant decreases in SMF as a result of 30-year accumulation of small and medium-sized plastic fragments, whereas the SMF remained relatively stable for large ones. The changes in soil ecosystem functions and bacterial communities markedly affected SMF through distinct pathways. Fragment size and bacterial community characteristics were identified as key variables. Key structural features of the network, such as network centrality nodes, network connectors and positive edge numbers, were examined as major driving factors influencing SMF. Also, the impacts of plastic fragments of different sizes on the bacterial network exhibited a complex pattern. Large plastic fragments had minimal effects on the basic network structure whereas small and medium-sized fragments decreased the network stability and were less concentration-dependent. It is worth noting that the sequencing method used in this study did not capture other microbial groups, such as fungi, archaea and protists. Despite this limitation, the data still show that uncollected polythene fragments to a large extent size-dependently altered SMF via mediating bacterial drivers in dryland. Our findings provided novel evidence for identifying the overall impacts of plastic fragments on SMF at the 30-year timescale.

## Supplementary Material

nwae281_Supplemental_File
