# Supplementary material for: Uncollected polythene fragments size-dependently alter soil multifunctionality via mediating bacterial drivers in dryland
Source: Natl Sci Rev. 2024 Aug 16;11(9):nwae281. doi: 10.1093/nsr/nwae281 (PMC11441323; doi:10.1093/nsr/nwae281)
Supplement: nwae281_Supplemental_File [file nwae281_supplemental_file.docx]

**Supplementary materials for**

**Uncollected polythene fragments size-dependently alter soil multifunctionality via mediating bacterial drivers in dryland**

Ze-Ying Zhao^a,1^, Peng-Yang Wang^a,1^, Xiao-Bin Xiong^a^, Yinglong Chen^b^, Hong-Yan Tao^a^, Wen-Ying Wang^c^, Yajie Song^d^, Muhammad Ashraf^e^, Li Zhu^f^, Yun-Li Xiao^f^, Shi-Sheng Li^f^, Fang-Kun Yang^a,c^, Meng-Ying Li^a^, Jing Cao^a^, Xiang-Wen Fang^a^, Levis Kavagi^g^, You-Cai Xiong^a,c^*

^a^ State Key Laboratory of Herbage Improvement and Grassland Agro-ecosystems, College of Ecology, Lanzhou University, Lanzhou 730000, China.

^b^ The UWA Institute of Agriculture, and School of Agriculture and Environment, The University of Western Australia, Perth 6001, WA, Australia.

^c^ Laboratory of Biodiversity Formation Mechanism and Comprehensive Utilization of the Qinghai-Tibet Plateau in Qinghai Province, Qinghai Normal University, Xining, 810008, China.

^d^ Global Institute of Eco-environment for Sustainable Development (GIESD), Yale School of the Environment, New Haven, CT 06511, USA.

^e^ Institute of Molecular Biology and Biotechnology, The University of Lahore, Lahore 54000, Pakistan.

^f^ College of Biology and Agricultural Resources, Huanggang Normal University, Huanggang 438000, China.

^g^ Division of Ecosystems and Biodiversity, United Nations Environment Programme, Nairobi 00100, Kenya.

*Corresponding author at State Key Laboratory of Herbage Improvement and Grassland Agro-ecosystems, College of Ecology, Lanzhou University, Lanzhou 730000, China.

E‒mail addresses: xiongyc@lzu.edu.cn (Y.C. Xiong) Tel/Fax.: +86-931-8914500

1. Z.Y. Zhao and P.Y. Wang contribute to this work equally.

**This** **file includes:**

Text S1

Figures S1 to S16

Table S1

**Text S1 Materials and methods**

***Site description***

Three-year (2019-2021) field experiment was conducted at the Modern Ecological Agriculture Innovation Station in the Hexi Corridor, Jinchang city of Gansu Province, China (38°15′N, 102°15′E, approximately 1430 m above sea level). The mean annual temperature is 9.4℃, and the mean annual precipitation is 241 mm (2011-2020). The site features a typical arid continental climate, with 85% of the rainfall occurring from June to September. The top layer of bulk soil (approximately 20 cm) has a pH of 8.3, soil bulk density of 1.48 g cm^-3^, soil organic carbon of 7.1 g kg^−1^, total nitrogen of 0.32 g kg^−1^, available phosphorus of 15.2 mg kg^−1^, and available potassium of 147.0 mg kg^−1^, respectively.

***Experimental design and sample collection***

As early as in 2012, a field survey indicated that the annual residue of plastic film in local farmland soil, amounted to approximately 45 kg ha^-1^. The data were provided by the local Agricultural Technology Extension Centre, suggesting a potential evasion of government supervision. Additionally, Yan et al. [1] found that in the 0-30 cm plough layer soils, the area of plastic film fragments fell into the scope between 1 and 2500 cm², with the main threshold from 4 to 25 cm^2^.

Based on the existing survey, we conducted field experiments and simulated three residual film sizes with two concentration gradients: small-sized (0.4×0.4 cm^2^), medium-sized (4×4 cm^2^), and large-sized (10×10 cm^2^), as well as two addition levels: low (L, 1350 kg ha^-1^, approximately 30 years of accumulation) and high (H, 2700 kg ha^-1^, approximately 60 years of accumulation). The three sizes of film residues conformed to the exponential function y=a*bx (where the initial term a is 0.16 and the common ratio b is 10). When y is 0.16, 16, and 100, the corresponding values of x are 0, 2, and 2.803 (approximately 3), respectively. In this case, a total of seven groups were created: (1) small_L, small-sized with low addition concentration; (2) medium_L, medium-sized with low addition; (3) large_L, large-sized with low addition; (4) small_H, small-sized with high addition concentration; (5) medium_H, medium-sized with high addition concentration; (6) large_H, large-sized with high addition concentration; (7) CK, no plastic residue addition.

The sizes of plastic fragments used in the experiment were created by cutting an equal mixture of transparent and black polyethylene films with sharp scissors. Before field preparation, plastic fragments that had accumulated in the 0-40 cm soil layer over the past few decades were sieved (using a pore size of 1 cm) and removed (Figure S1). The experiment was arranged with three replicates. Each plot was arranged in an area of 4 m × 5 m. Plastic fragments were uniformly spread across the plots and subsequently plowed into the soil to a depth of 20 cm. Following local agricultural practices, the basal fertilizer application rates were 205 kg N ha^-1^, 108 kg P ha^-1^, and 50 kg K ha^-1^. No additional fertilization was applied during the growing season. This three-year field experiment, starting in 2019, included three maize growing seasons. The sowing dates were April 26, 2019; April 24, 2020; and May 13, 2021, with corresponding harvest dates of September 28, 2019; October 7, 2020; and October 18, 2021. Maize variety *Xianyu1225* was planted in traditional flat cropping. All the treatments were covered with black plastic film. Field irrigation and pest and disease control practices were performed in accordance with local agricultural conventions.

At the physiological maturity, five random maize plants were selected in each plot to measure biomass, four representative and undamaged planted rows were selected in each plot to measure maize yield. Maize biomass and yield were continuously monitored for three years. After maize harvest on October 18, 2021, three soil samples were randomly collected from each sampling plot at a depth of 0-20 cm using soil auger (diameter, 5.0 cm). Soil samples from each plot were combined into composites, mixed evenly and then quickly divided into three parts. One part was naturally air-dried and sieved (2 mm and 0.15 mm). A small part of the soil (~25 g) for DNA extraction was stored at -80 ℃, and the remaining part was stored at -20 ℃ (2 mm). The undisturbed soil samples (0-20 cm) were harvested using the stainless-steel cylindrical cores (8 cm inner diameter, 20 cm height) to determine soil water-stable aggregates.

***Sample analysis***

Soil pH was assessed using a pH electrode with a soil-to-water ratio of 1:2.5. The method involving potassium dichromate-concentrated sulfuric acid external heating oxidation was employed for soil organic carbon (SOC) measurement. Soil total nitrogen (TN) was determined using the Kjeldahl method-automatic, while soil ammonium nitrogen (NH_4_^+^-N) and nitrate nitrogen (NO_3_^-^-N) were quantified using the Skalar San++ analyzer (the Netherlands). Microbial biomass carbon (MBC) and microbial biomass nitrogen (MBN) were assessed using the CHCl₃ fumigation-extraction method. Dissolved organic carbon (DOC) was extracted with K_2_SO_4_ and analyzed by a multi-N/C analyzer. Easily oxidizable organic carbon (EOC) was determined through the KMnO_4_ oxidation method. Soil urease activity was measured using urea as a substrate, while soil β-glucosidase activity was assessed with p-nitrophenyl-β-D-glucopyranoside as the substrate [2]. Soil temperature between two plants in each treatment plot row was recorded hourly throughout the maize growing season at a depth of 15 cm, spanning from 0:00 to 23:00 each day. Soil bulk density was determined by oven-drying soil cores at 105 °C until reaching a constant weight. Soil porosity was calculated by measuring soil bulk density and volumetric water content. Separation of water-stable aggregates was performed through the combined sieves of 2 mm/1 mm/0.5 mm/0.25 mm/0.1 mm.

***Amplicon sequencing***

Genomic DNA was extracted from 0.5 g of soil using the Soil DNA Isolation Kit (TIANGEN DP336, China), following the manufacturer's protocols. The bacterial 16 S rRNA gene was amplified using primers 515 F (5'-GTGCCAGCMGCCGCGGTAA-3') and 806 R (5'-GGACTACHVGGGTWTCTAAT-3') targeting the hypervariable V4-V5 regions. Sequencing was performed on the Illumina HiSeq 2500 platform (Illumina, USA). The obtained sequencing data have been deposited in the NCBI database.

***Assessing soil multifunctionality***

To assess soil multifunctionality, we focused on five essential soil functions, encompassing 12 variables crucial to soil biogeochemical processes and ecosystem functions [3,4]. These functions included nitrogen stocks (represented by soil TN, NH_4_^+^-N, and NO_3_^-^-N; Figure S2), carbon stocks (evaluated by SOC, DOC, and EOC; Figure S3), enzyme activity (indicated by soil enzyme activities URE and BG; Figure S4), crop production (measured through grain yield, aboveground biomass; Figure S5), and DNA concentration (assessed by MBC and MBN; Figure S6) [5]. For maize biomass and yield indicators, the averages of monitoring data collected between 2019 and 2021were used. However, the soil data were specifically obtained in 2021. All 12 variables were standardized using Z-score transformation. The standardized means of the variables within the above groups are used to calculate the 5 functional groups (nitrogen stocks, nitrogen stocks, enzyme activity, crop production, DNA concentration), and the standardized means of the 12 variables are used to evaluate the SMF [3].

***Statistical analyses***

Two-factor analysis of variance (ANOVA) was employed to investigate the impacts of plastic fragment sizes and concentrations on soil functions, including sole functions, multifunctionality, bacterial diversity, bacterial abundance, bacterial functional diversity, and co-occurrence network parameters. Bacterial diversity and bacterial functional diversity were assessed using the Shannon index. Bacterial richness was represented by the ACE index (Abundance-based Coverage Estimator metric). Principal component analyses (PCA) were performed to reduce dimensionality and visualize the soil functional groups. The differences in multiple soil functions under the plastic fragment exposure were compared using permutational multivariate analysis of variance (PERMANOVA) with the Adonis function in the “vegan” package in R. On the other hand, Pearson's correlation analyses were performed to examine the relationships between soil bacterial community characterization (bacterial diversity, bacterial abundance, bacterial functional diversity), soil multifunctionality (SMF), and the connections between sole soil functions. Ordinary least squares (OLS) linear regression models were constructed to explore the relationships among biotic and abiotic factors, sole soil functions, soil bacterial community characterization, and SMF.

Also, Piecewise structural equation modeling (PiecewiseSEM) was implemented, incorporating existing knowledge on soil bacterial community characterizations, soil functional groups, and SMF interactions to assess the association between bacterial community characterizations with SMF while considering multiple environmental factors. Soil pH, playing a crucial role in nutrient cycling and availability, was included solely for SEM analysis due to its logarithmic scale. Plastic fragment size (categorized as small-sized, 1; medium-sized, 2; large-sized, 3) and concentration (categorized as low, 1; high, 2) were set as categorical exogenous variables. A within-group Bootstrap method was used to ensure the establishment of the SEM [8]. Following the approach by Liu et al. [9], all measured variables were first grouped into 'composite variables' before being incorporated into the SEM (Fig. 1G). These analyses were conducted using "piecewiseSEM", "nlme" and "lme4" packages in R [9]. Fisher’s C-test (when 0.05<*p*<1.00) was used to confirm the goodness of the modelling results. Note that we only colored in the significant relationships (two-sided *p*<0.25) and displayed their coefficients (numbers adjacent to arrows) for graphical simplicity. The total effect of a variable on SMF was determined by considering all direct and indirect pathways between the two.

For the construction of co-occurrence networks, bacterial taxa with a relative abundance greater than 0.1% were selected. Only nodes that were significantly correlated each other (spearman’s>0.7, *p*<0.05) were connected, and only nodes with these connections were retained. The adjacency matrix encompassing all treatment groups was initially constructed, and subnetworks were extracted by preserving the community fingerprints of individual soil samples [8]. Network topological characteristics for each sample and Zi (within-module connectivity)-Pi (among-module connectivity) plots were generated using the “igraph” package in R. The threshold values of Zi and Pi for categorizing ASVs were set at 2.5 and 0.62, respectively [6]. Visualization of the co-occurrence network was completed using Gephi v.0.9.6. We constructed a random forest model with 1000 decision trees using the randomForest R package to identify major significant predictors for SMF, and assessed the significance of each predictor's importance using the rfPermute R package. These predictors included soil bacterial community characterizations, soil functional groups, and the topological roles of the bacterial co-occurrence network (e.g., numbers of positive edge, negative edge, nodes, average degree, average clustering coefficient, average path length, connectors, module hubs, and network hubs) [7]. To ensure the robustness and generalizability of the model, we employed Leave-One-Out Cross-Validation (LOOCV) with 1000 decision trees. With the target variable (SMF) ranging from 0-1, the model's cross-validated RMSE (root mean squared error) was 0.054, the R^2^ was 0.722, and the MAE (mean absolute error) was 0.048. These results indicate that the model has good predictive performance and explanatory power. It is noteworthy that the R^2^ of model is based on cross-validation result, providing a more reliable estimate of the model's generalization performance.

**Reference**

1. Yan C, Liu E, Shu F, et al. Review of agricultural plastic mulching and its residual pollution and prevention measures in China. Journal of Agricultural Resources and Environment, 31, 95-102 (2014). (in Chinese with English abstract)
2. Zhao Z, Wang P, Xiong X, et al. Environmental risk of multi-year polythene film mulching and its green solution in arid irrigation region. Journal of Hazardous Materials, 435, 128981 (2022).
3. Wang J, Shi X, Lucas‐Borja M E, et al. Soil nematode abundances drive agroecosystem multifunctionality under short‐term elevated CO2 and O3. Global Change Biology, 29, 1618-1627 (2023).
4. Li J, Delgado-Baquerizo M, Wang J T, et al. Fungal richness contributes to multifunctionality in boreal forest soil. Soil Biology and Biochemistry, 136, 107526 (2019).
5. Gong H, Du Q, Xie S, et al. Soil microbial DNA concentration is a powerful indicator for estimating soil microbial biomass C and N across arid and semi-arid regions in northern China. Applied Soil Ecology, 160, 103869 (2021).
6. Garre A, Fernández PS, Truchado P, et al. The use of Bayesian networks and bootstrap to evaluate risks linked to the microbial contamination of leafy greens irrigated with reclaimed water in Southeast Spain. Microbial Risk Analysis, 22, 100234 (2022).
7. Liu S, García-Palacios P, Tedersoo L, et al. Phylotype diversity within soil fungal functional groups drives ecosystem stability. Nature Ecology & Evolution, 6, 900-909 (2022).
8. Zhu L, Luan L, Chen Y, et al. Community assembly of organisms regulates soil microbial functional potential through dual mechanisms. Global Change Biology, 30, e17160 (2024).
9. Qiu L, Zhang Q, Zhu H, et al. Erosion reduces soil microbial diversity, network complexity and multifunctionality. The ISME journal, 15, 2474-2489 (2021).
10. Liaw A, Wiener M. Classification and regression by randomForest. R news, 2, 18-22 (2002).


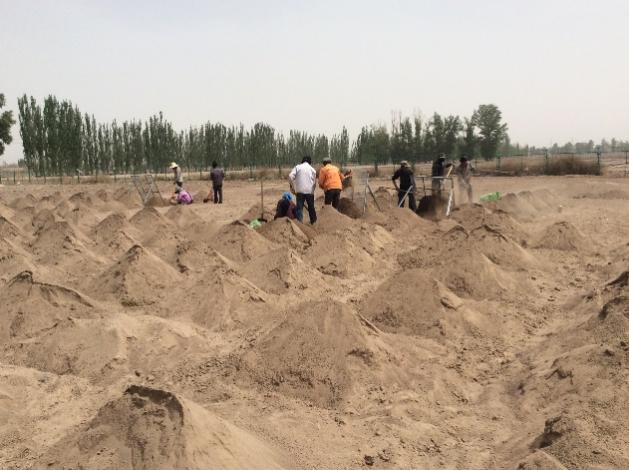

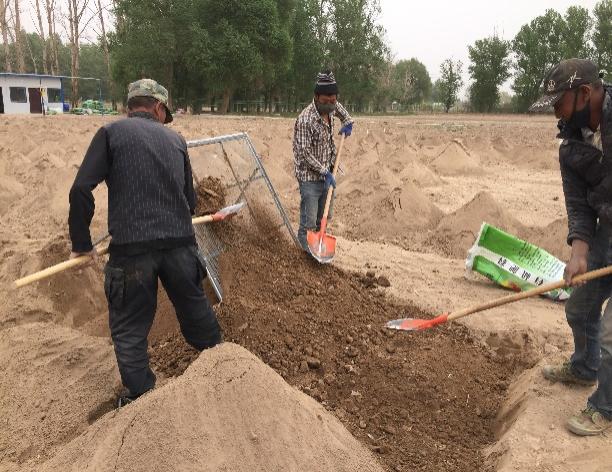


**Figure S1. The waste plastic film in the 0-40 cm soil layer was sieved and removed before landfill experiment.**





**Figure S2. The effects of** **plastic fragment contamination on soil total nitrogen, nitrate nitrogen and ammonium nitrogen.** L, low addition amount. H, high addition amount. S, size of plastic fragments; C, concentration of plastic fragments. Different lower-case letters indicate the significant differences at *p*<0.05. ***, *p*<0.001; **, at *p*<0.01; *, *p*<0.05 (the same for the below figures).





**Figure S3. The effects of plastic fragment contamination on soil urease activity and β-glucosidase activity.**





**Figure S4. The effects of plastic fragment contamination on microbial biomass carbon and microbial biomass nitrogen.**





**Figure S5. The effects of plastic fragment contamination on maize biomass and maize yield from 2019 to 2021.**





**Figure S6. The effects of plastic fragment contamination on soil organic carbon, dissolved organic carbon and easily oxidizable carbon.**


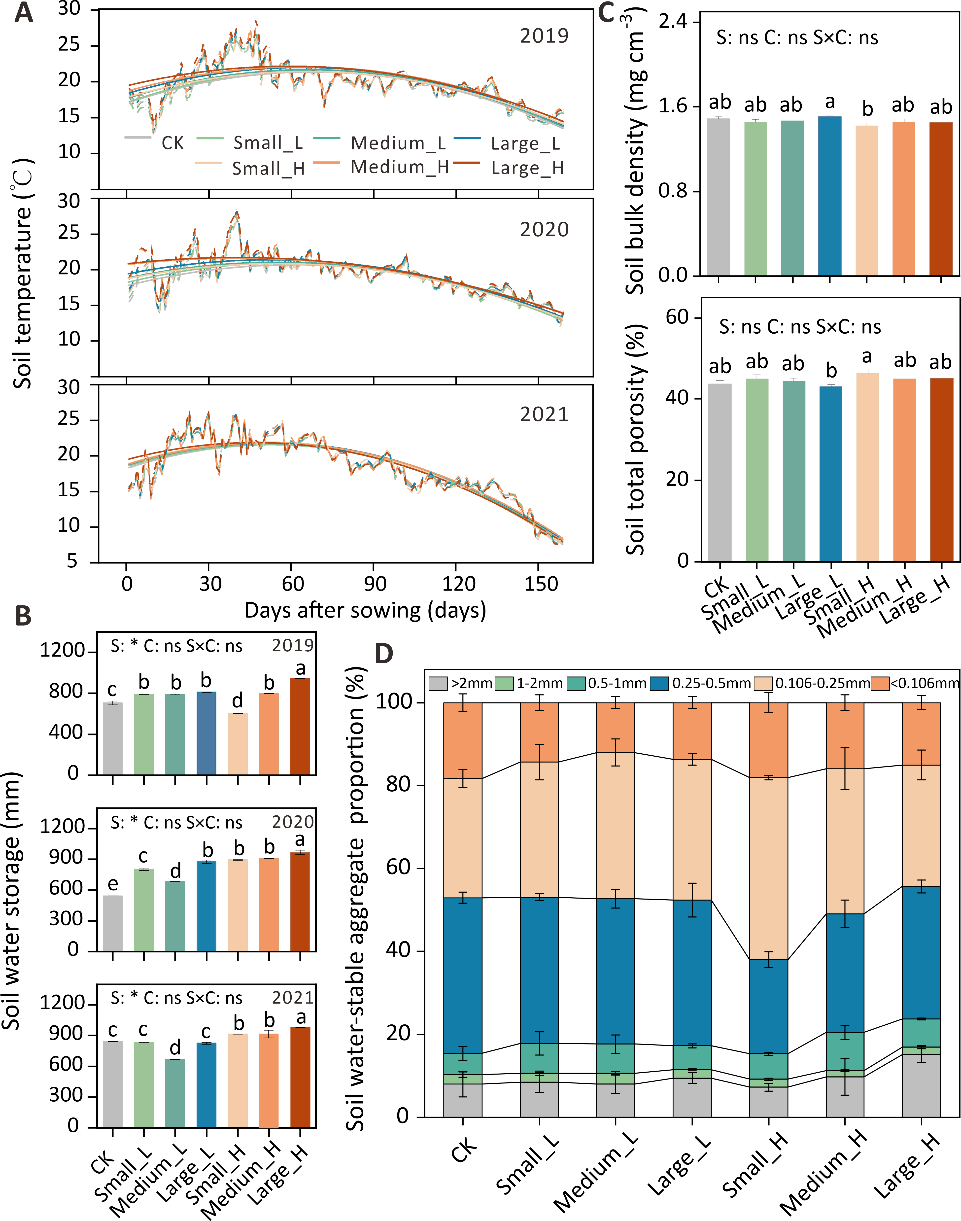


**Figure S7 The effects of plastic fragment contamination on soil hydrothermal state and physical structure.** (A) Soil temperature in 2019-2021. (B) Soil water storge in 2019-2021. (C)Soil bulk density and soil total porosity. (D) Soil water-stable aggregate proportion.


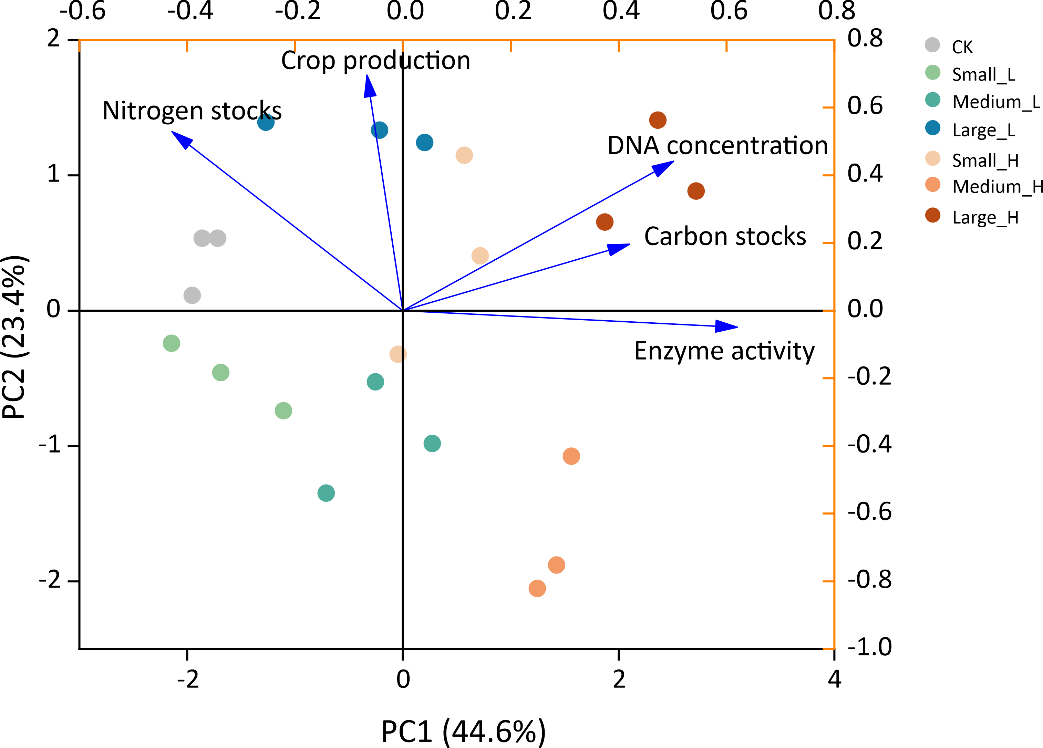


**Figure S8. The structure of multiple ecosystem functions analyzed by principal component analyses (PCA) analysis.**


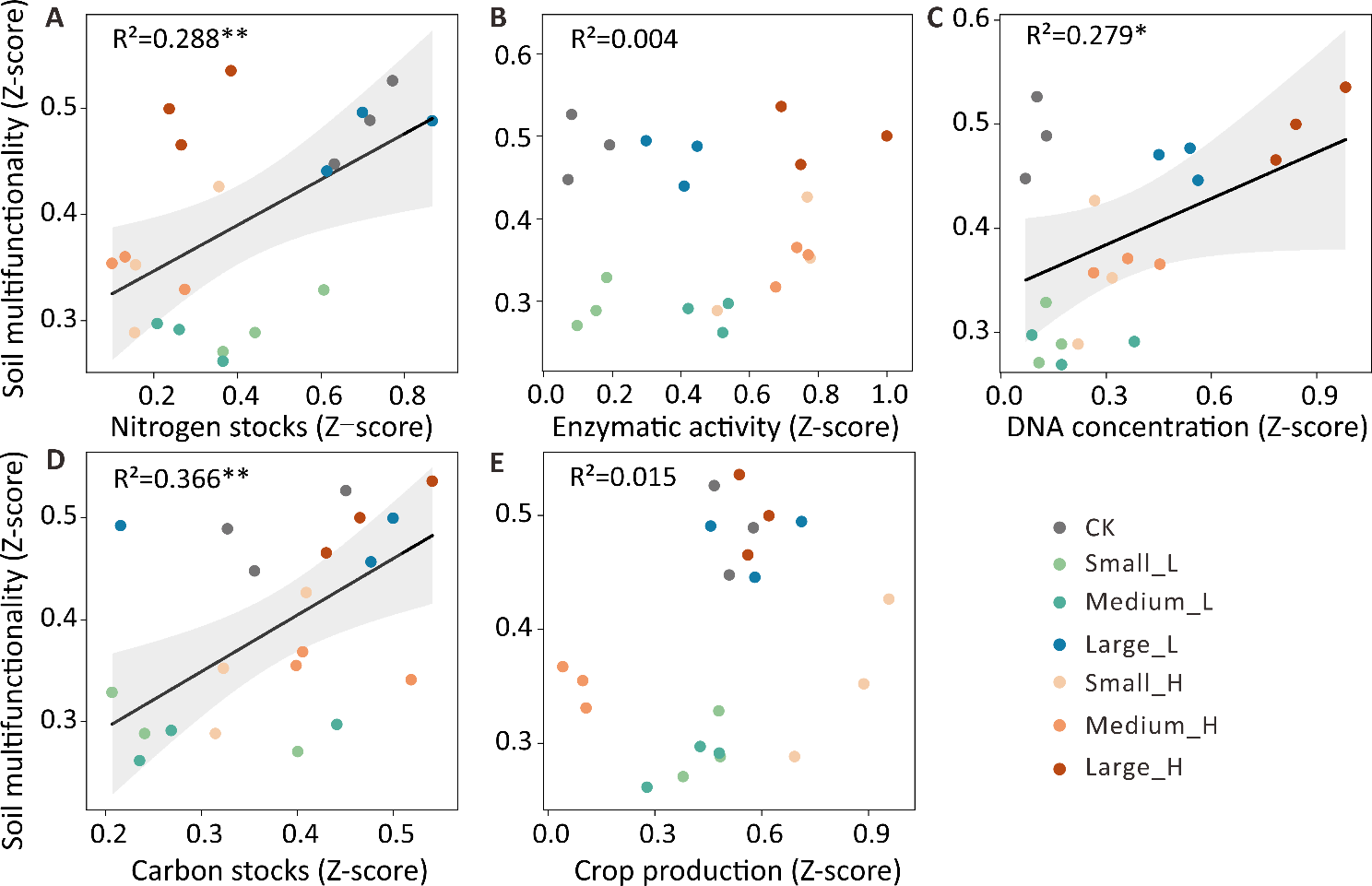


**Figure S9. The correlation between multiple ecosystem functions and soil multifunctionality.** Nitrogen stocks (represented by soil TN, NH_4_^+^-N, and NO_3_^-^-N), Enzymatic activity (including soil urease activity and β-glucosidase activity), DNA concentration (assessed by MBC and MBN), Carbon storage (evaluated by SOC, DOC, and EOC), and Crop production (measured through grain yield and biomass).


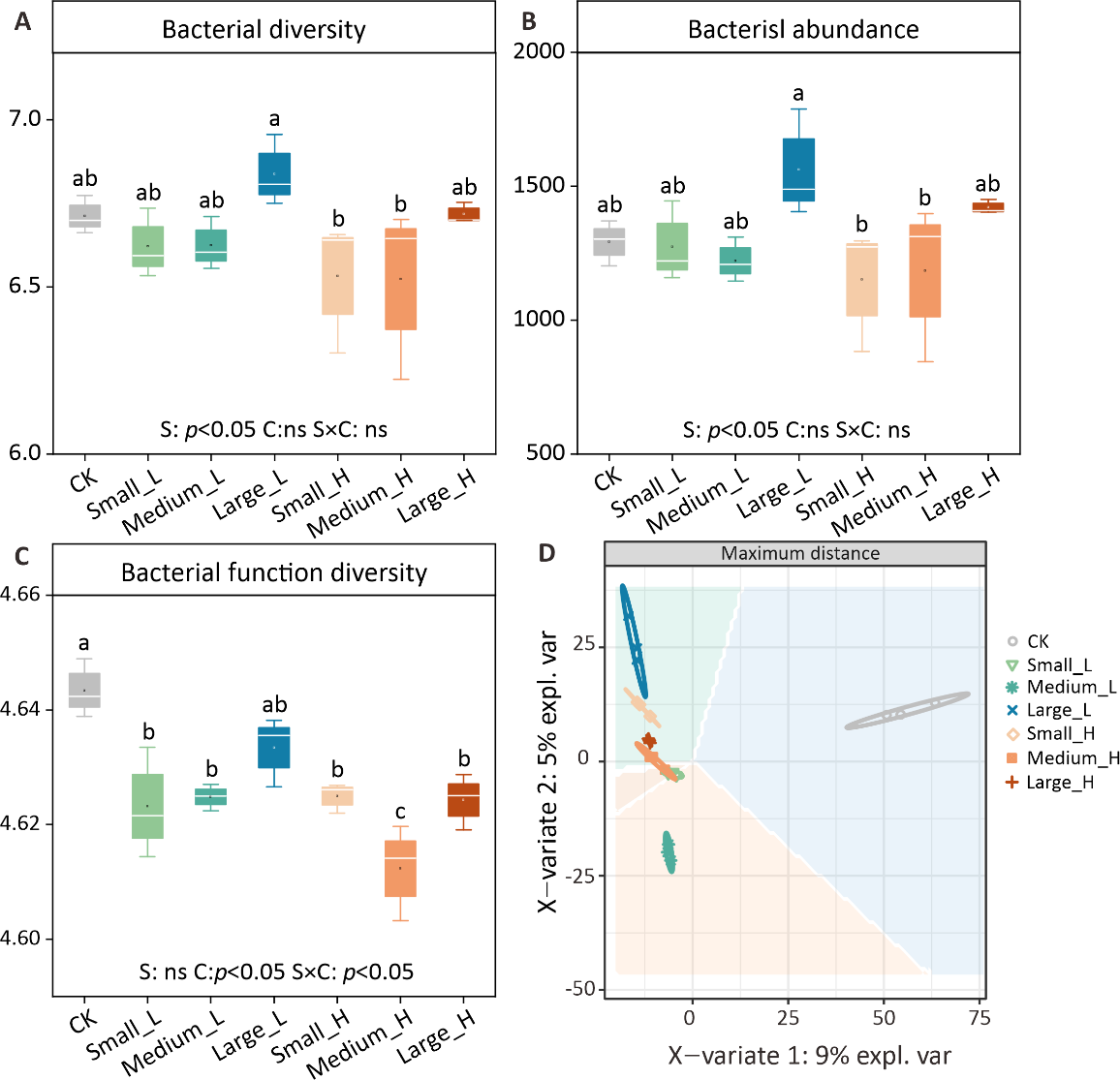


**Figure S10. The** **plastic fragment pollution effects on soil bacterial diversity and composition.** (A) Diversity of the bacterial communities (Shannon index). (B) Bacterial abundance (Abundance-based coverage estimator index). (C) Bacterial functional diversity (Shannon index). (D) Principal coordinate analysis (PLS_DA) based on the Bray-Curtis distance indices of the bacterial communities.


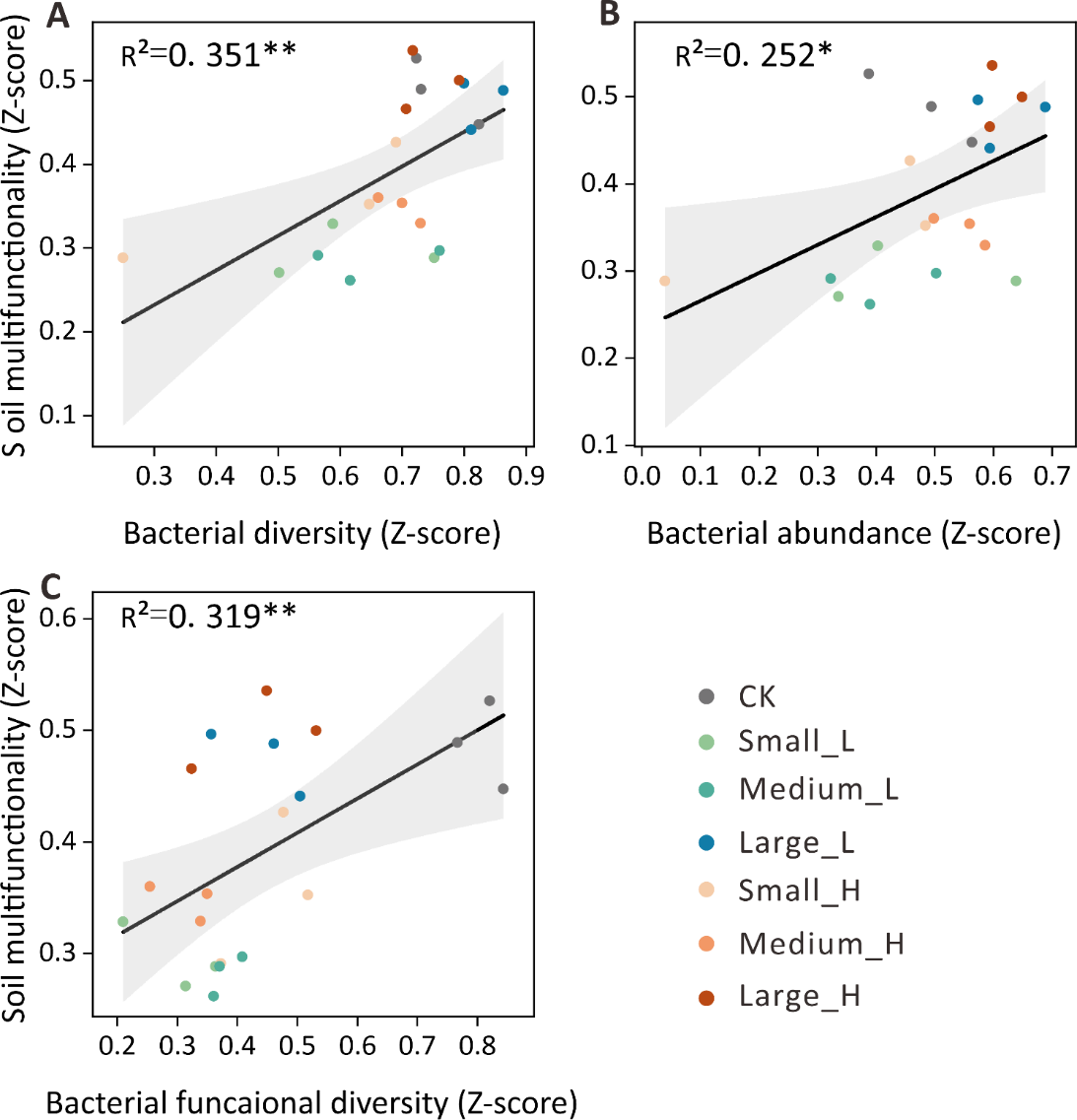


**Figure S11. The correlation between bacterial diversity (A), bacterial abundance (B) and bacterial functional diversity (C) and soil multifunctionality.**


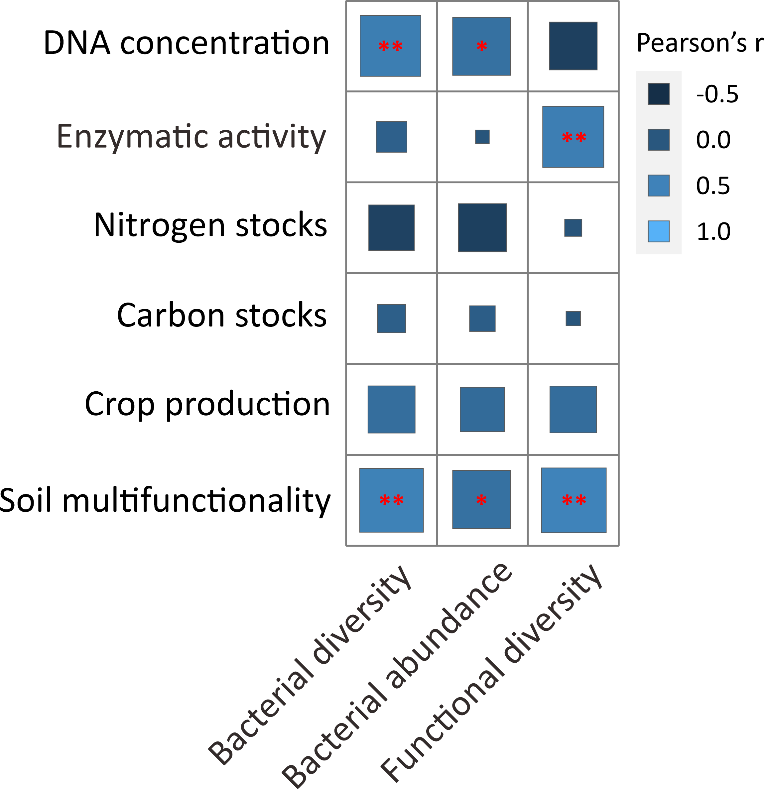


**Figure S12. Correlation and predictive analyses of soil multifunctionality with biotic and abiotic factors.** (A) The correlation between multiple soil functional index and soil bacterial community characterization (bacterial diversity, bacterial abundance and functional diversity). The color presents the Pearson's correlation (The lighter the blue, the higher the positive correlation). (B) Main predictors of soil multifunctionality characterized by random forest modelling analysis. The figure shows the random forest results with main predictor importance (% of increase of MSE) of sole soil function and soil bacterial community characterization on soil multifunctionality.


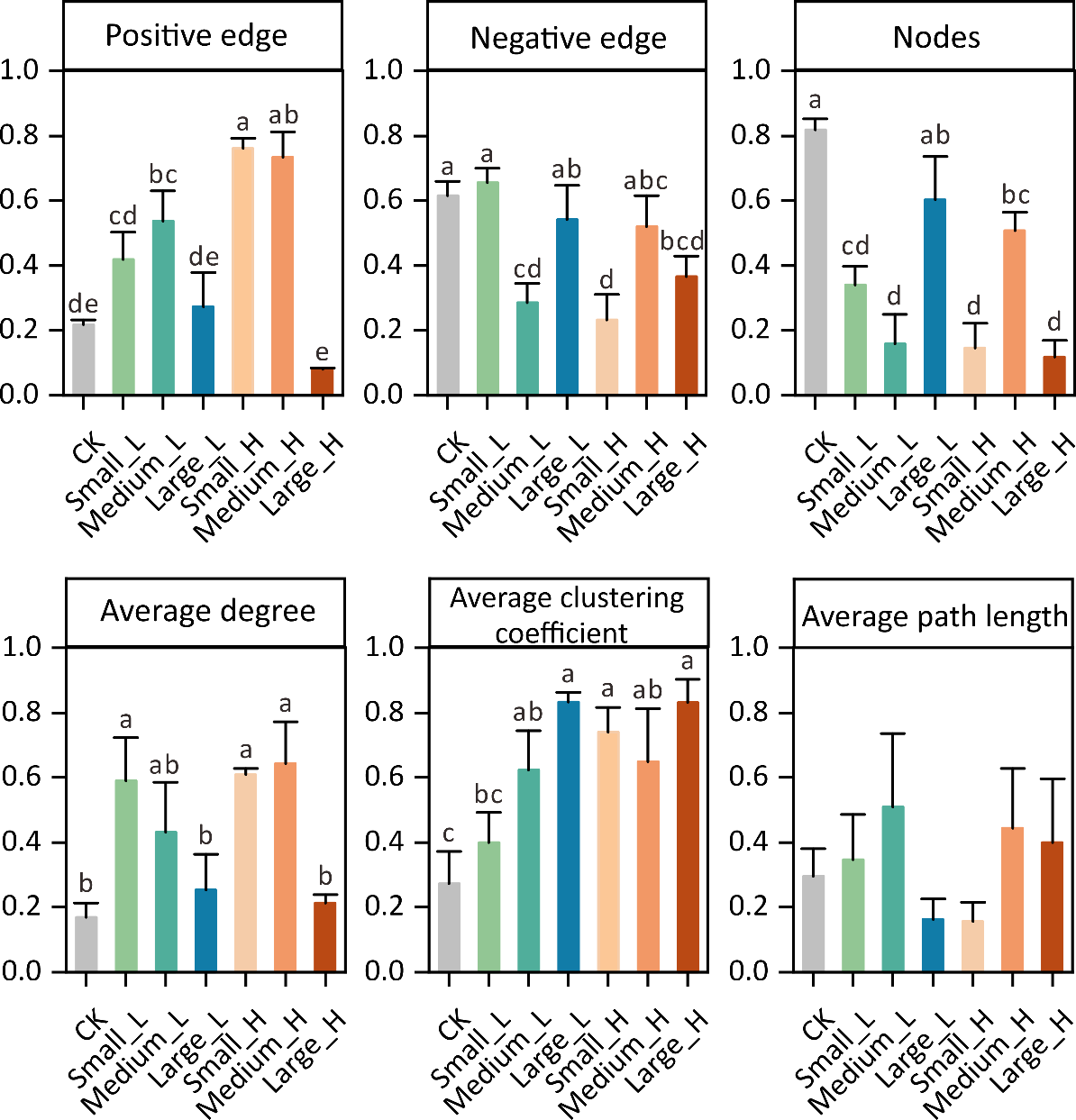


**Figure S13. Co-occurrence patterns in soil bacterial network as affected by plastic fragments pollution.** The responses of the numbers of positive edge, negative edge, nodes, average degree, average clustering coefficient and average path length to plastic fragments pollution.


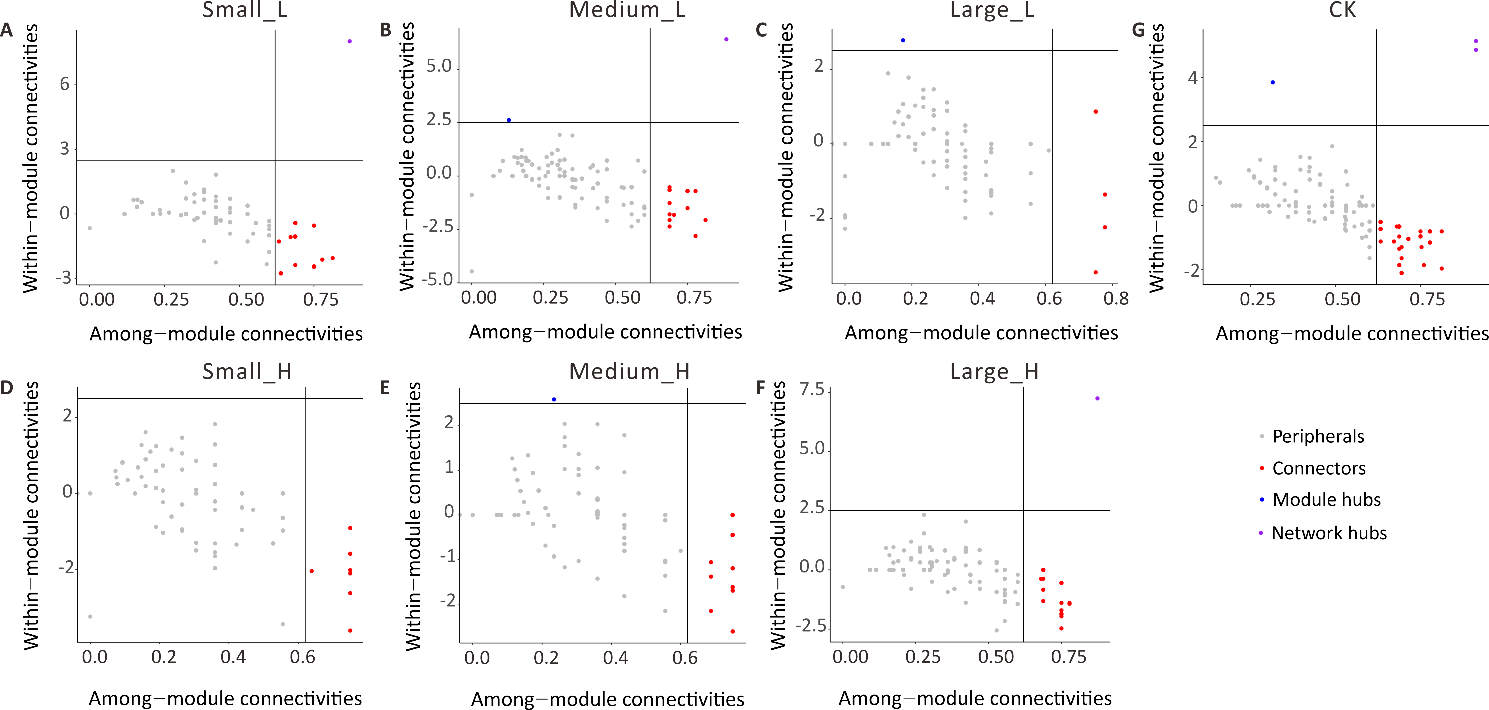


**Figure S14. Zi-Pi plots and keystone species under different residual plastic film.** The threshold values of Zi and Pi for categorizing ASVs were 2.5 and 0.62, respectively. Peripherals (Zi<2.5, Pi<0.62), module hubs (Zi≥2.5, Pi<0.62), connectors (Zi<2.5, Pi>0.62), network hubs (Zi≥2.5, Pi≥0.62).


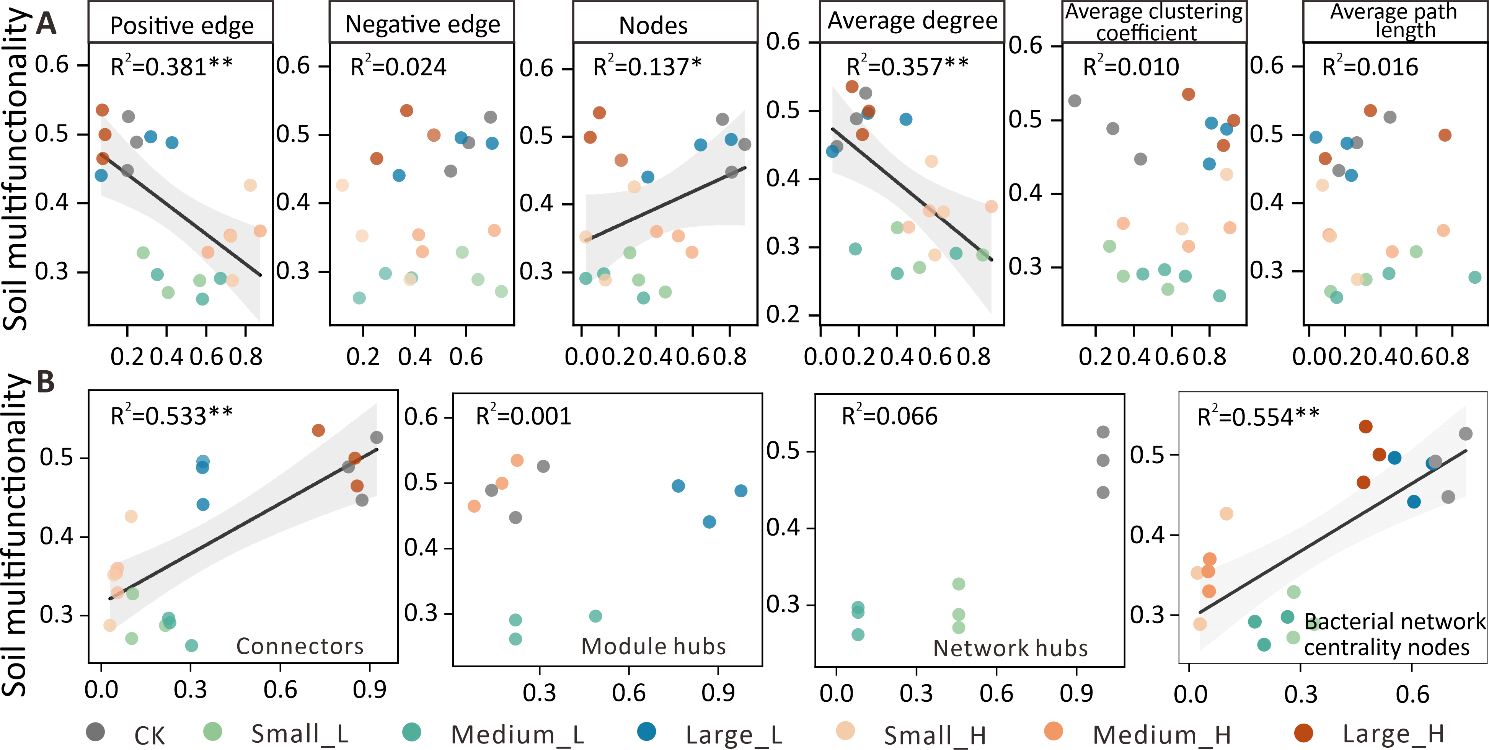


**Figure S15. Correlations between the topological roles of ASVs and soil multifunctionality.** (A) The relationships of soil multifunctionality to network topological characteristics (including numbers of positive edge, negative edge, nodes, average degree, average clustering coefficient and average path length of soil bacteria co-occurrence patterns). (B) The relationships of soil multifunctionality to nodes topological characteristics，connectors, module hubs, and network hubs of soil bacteria co-occurrence patterns).


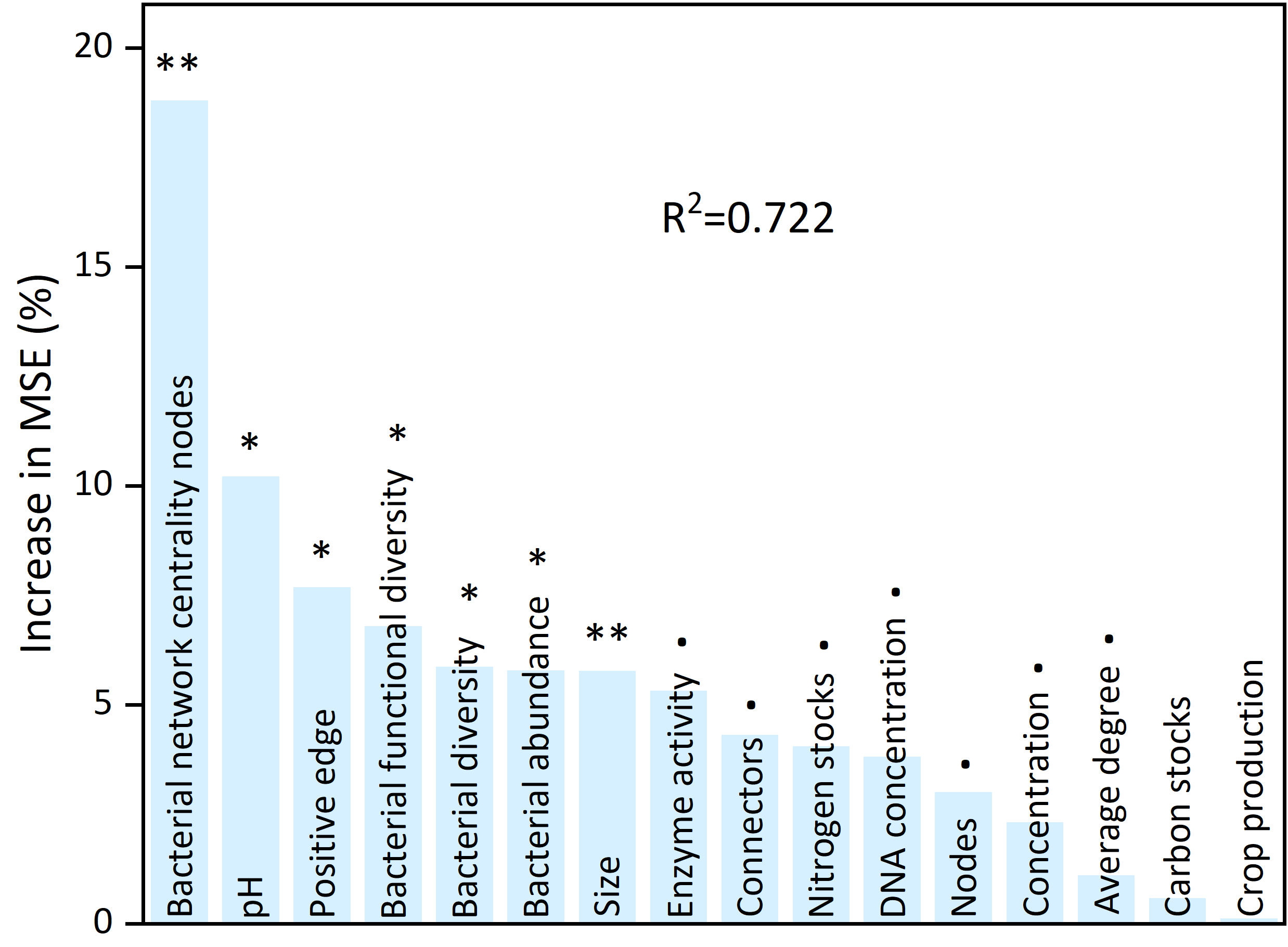


**Figure S16. Random forest regression model shows the main topological roles drivers of soil multifunctionality.**

**Table S1 Tests of fixed effects for the two factors and the factor interactions on soil bacterial co-occurrence patterns.**

| Factors | Positive edge | Negative edge | Nodes | Average degree | Average clustering coefficient | Average path length |
| --- | --- | --- | --- | --- | --- | --- |
| Size | * | ns | * | ** | ns | ns |
| Concentration | ns | ns | ns | ns | ns | ns |
| Size × Concentration | *** | *** | *** | ns | ns | ns |

Note: *Significant at *p*<0.05; **Significant at *p*<0.01; ***Significant at *p*<0.001; ns means no significant
